# Reduction of *Stabilin-2* Contributes to a Protection Against Atherosclerosis

**DOI:** 10.3389/fcvm.2022.818662

**Published:** 2022-03-11

**Authors:** Yukako Kayashima, Connor A. Clanton, Amanda M. Lewis, Xinghui Sun, Sylvia Hiller, Phillip Huynh, Jennifer Wilder, John Hagaman, Feng Li, Nobuyo Maeda-Smithies, Edward N. Harris

**Affiliations:** ^1^Department of Pathology and Laboratory Medicine, University of North Carolina at Chapel Hill, Chapel Hill, NC, United States; ^2^Department of Biochemistry, University of Nebraska, Lincoln, NE, United States

**Keywords:** mouse, aorta, atherosclerosis, hyaluronic acid, stabilin 2

## Abstract

We have previously identified a novel atherosclerosis quantitative trait locus (QTL), *Arch atherosclerosis 5* (*Aath5*), on mouse chromosome 10 by three-way QTL analyses between *Apoe*^−/−^ mice on a DBA/2J, 129S6 and C57BL/6J background. The DBA/2J haplotype at the *Aath5* locus was associated with smaller plaque size. One of the candidate genes underlying *Aath5* was *Stabilin-2* (*Stab2*), which encodes a clearance receptor for hyaluronan (HA) predominantly expressed in liver sinusoidal endothelial cells (LSECs). However, the role of *Stab2* in atherosclerosis is unknown. A congenic line of *Apoe*^−/−^ mice carrying *Aath5* covering the *Stab2*^*DBA*^ allele on a background of 129S6 confirmed the small reductions of atherosclerotic plaque development. To further determine whether *Stab2* is an underlying gene for *Aath5*, we generated *Stab2*^−/−^*Apoe*^−/−^ mice on a C57BL/6J background. When fed with a Western diet for 8 weeks, *Stab2*^−/−^*Apoe*^−/−^ males developed approximately 30% smaller plaques than *Stab2*^+/+^*Apoe*^−/−^ mice. HA was accumulated in circulation but not in major organs in the *Stab2* deficient mice. STAB2-binding molecules that are involved in atherosclerosis, including acLDL, apoptotic cells, heparin and vWF were not likely the direct cause of the protection in the *Stab2*^−/−^*Apoe*^−/−^ males. These data indicate that reduction of *Stab2* is protective against atherosclerotic plaque development, and that *Stab2* is a contributing gene underlying *Aath5*, although its effect is small. To test whether non-synonymous amino acid changes unique to DBA/2J affect the function of STAB2 protein, we made HEK293 cell lines expressing STAB2^129^ or STAB2^DBA^ proteins, as well as STAB2^129^ proteins carrying each of five DBA-unique replacements that have been predicted to be deleterious. These mutant cells were capable of internalizing ^125^I -HA and DiI-acLDL similarly to the control cells. These results indicate that the amino acid changes unique to DBA/2J are not affecting the function of STAB2 protein, and support our previous observation that the reduced transcription of *Stab2* in the liver sinusoid as a consequence of the insertion of a viral-derived sequence, intracisternal A particle, is the primary contributor to the athero-protection conferred by the DBA/2J allele.

## Introduction

Atherosclerosis, which accounts for more than 30% of all deaths worldwide, is a complex multifactorial disease (1). Well-known risk factors of atherosclerosis include hyperlipidemia, hypertension, diabetes and chronic inflammation, as well as lifestyle risk factors such as smoking, high calorie/nutritionally poor diets and lack of physical activity. Many genetic factors additively or interactively determine susceptibility to atherosclerosis of an individual, although the genetic factors involved in the pathogenesis are not fully understood (2, 3).

Mouse models of atherosclerosis with different genetic backgrounds show clear evidence of genetic factors affecting susceptibility to plaque development (4, 5). For example, atherosclerotic plaque size of apolipoprotein E-deficient (*Apoe*^−/−^) mice on a DBA2/J background (DBA-*Apoe*^−/−^) is larger than those on a C57BL/6J background (B6-*Apoe*^−/−^) or a 129S6 background (129-*Apoe*^−/−^) (6–8). To identify the genetic factors influencing the plaque size, we previously performed quantitative trait locus (QTL) analysis using the F2 population from DBA-*Apoe*^−/−^ x 129-*Apoe*^−/−^, and identified two new QTLs, *Aath4* on Chromosome (Chr) 2 and *Aath5* on Chr 10 (7). Analyses of plaque size in the F2 population revealed that the DBA allele of *Aath4* confers susceptibility for plaque development in the aortic arch area, whereas the DBA allele at *Aath5* is protective. The atherosclerosis-enhancing effect of *Aath4* likely overrides the protective effect of *Aath5*, since DBA/2J strain is more susceptible to atherosclerosis compared to B6 and 129. We further showed that *Mertk* is a responsible gene underlying *Aath4* (9).

*Aath5* was also detected by a QTL analysis using a cross between DBA-*Apoe*^−/−^ and B6-*Apoe*^−/−^, while it was not present in 129-*Apoe*^−/−^ x B6-*Apoe*^−/−^ (8, 10). Collectively, these results indicate that the DBA/2J allele of *Aath5* is unique, whereas 129S6 and C57BL/6 alleles share the same sequences. Comparison of *Aath5* haplotypes between DBA/2J, 129S6 and C57BL/6 revealed that only a few genes in this region exhibit this pattern. Among those genes, *Stabilin2* (*Stab2*) has potentially harmful amino acid substitutions, as well as differential mRNA expression levels in macrophages and aortic tissues (7). Although no SNPs relevant to atherosclerosis, or notable cis- or trans-expression QTLs were detected within the interval in the mouse genome, variations near or within the human *STAB2* gene are associated with coronary restenosis (*p* = 1.0 × 10^−7^), coronary disease (*p* = 1.0 × 10^−5^) and stroke (*p* = 5.4 × 10^−4^). Thus, *Stab2* is a potential candidate for *Aath5*.

*Stab2* encodes a scavenger receptor primarily expressed in the sinusoidal endothelial cells (SECs) of the liver, spleen, bone marrow and lymph nodes. Many ligands are known to bind STAB2, including hyaluronic acid (HA), heparins, chondroitin sulfates, modified LDLs, collagen, von Willebrand factor/Factor VIII and advanced glycation end products (11–15). We previously reported that plasma concentration of HA is ten-fold higher in DBA/2J mice than in 129S6 and C57BL/6J mice, and that the difference is linked to the *Stab2* locus but not to the other gene loci involved in HA metabolism, such as HA synthases, hyaluronidases and other receptors (7). We further showed that the promoter region of *Stab2* in the DBA/2J genome contains a viral-derived sequence, intracisternal A particle (IAP), and that *Stab2* gene expression is lower in the liver sinusoidal endothelial cells (LSECs) of DBA/2J mice compared to 129 mice, suggesting that the reduced expression of *Stab2* could lead to the high plasma HA levels in DBA/2J mice (16).

*Stab2* is reported to be expressed in the atherosclerotic plaques in humans, although the role of *Stab2* in atherosclerosis is unclear (17). Knockout mice of *Stab2* exhibit more than 10 times higher plasma HA than the wild-type mice, but otherwise are grossly normal (18). Schledzewski et al. demonstrated that double knockout mice of *Stab1* and *Stab2* show glomerulofibrotic nephropathy due to accumulation of a transforming growth factor (TGF)-β family member growth differentiation factor 15 (GDF-15) and other ECM material in the blood, while single knockout mice of *Stab2* are normal (18). The double knockout phenotype also leads to placental deficiencies that substantially decrease litter numbers not seen in single *Stab1* or *Stab2* KO mice (19). Hirose *et al*. showed that tumor metastasis was prevented in *Stab2* knockout mice (20). However, there is no investigation directly evaluating that *Stab2* is involved in atherosclerosis. Finally, although the major ligand of STAB2, HA, is implicated in atherosclerosis, HA could be protective or harmful depending on the context (21–24).

In this study, we demonstrated that *Aath5* affects atherosclerosis by establishing an *Apoe*^−/−^ line on a 129S6 background that are homozygous for *Aath5* of DBA/2J allele, and further examined the role of *Stab2* in atherosclerosis by generating *Stab2*^−/−^*Apoe*^−/−^ mice on a C57BL/6J background.

## Materials and Methods

### Animals

*Apoe*^−/−^ mice on C57BL/6J and 129S6/SvEvTac backgrounds were generated in our laboratory as previously described (5). Mice were fed regular mouse chow (3002909-203, PicoLab) and handled under protocols approved by the Institutional Animal Care and Use Committees of the University of North Carolina at Chapel Hill and the University of Nebraska. Under specific nutritional protocols, the mice were also fed a Western-type diet (42% calories from fat, TD88137, Harlan Teklad).

### Generation of a Congenic *Aath5^*DBA*/*DBA*^* Line on a 129S6 Background

We have established *Aath5*^*DBA*/*DBA*^ congenic mice by continuously backcrossing Chr 10: 86 Mb (*Aath5*) of DBA/2J onto the 129S6 (Taconic Biosciences) background for ten generations. The DBA allele was discriminated from the 129 allele by PCR using primers that detect a fragment length polymorphism within the intron 1 of *Stab2* gene: 5'-CTGAGAAACAGGTGGCATGT-3' and 5'-TATGCCTGCCTGACGGATTA-3'. The 129 allele produces a 190 bp band while the DBA allele produces a 170 bp band. A heterozygous mouse at the tenth backcross generation was then crossed with 129-*Apoe*^−/−^ mice. Heterozygous mice were further intercrossed to generate homozygous *Aath5*^*DBA*/*DBA*^ mice. The extent of the DBA based genome were determined to be 84.6 Mb to 93.9 Mb using SNP genotyping Mouse Universal Genotyping Arrays (GigaMUGA) with a help from the UNC Systems Genetics Core (25). The *Aath5*^*DBA*/*DBA*^ mice were fed regular chow (3002909-203, PicoLab).

### Generation of *Stab2^−/−^Apoe^−/−^* Mice

**Figure 1:**
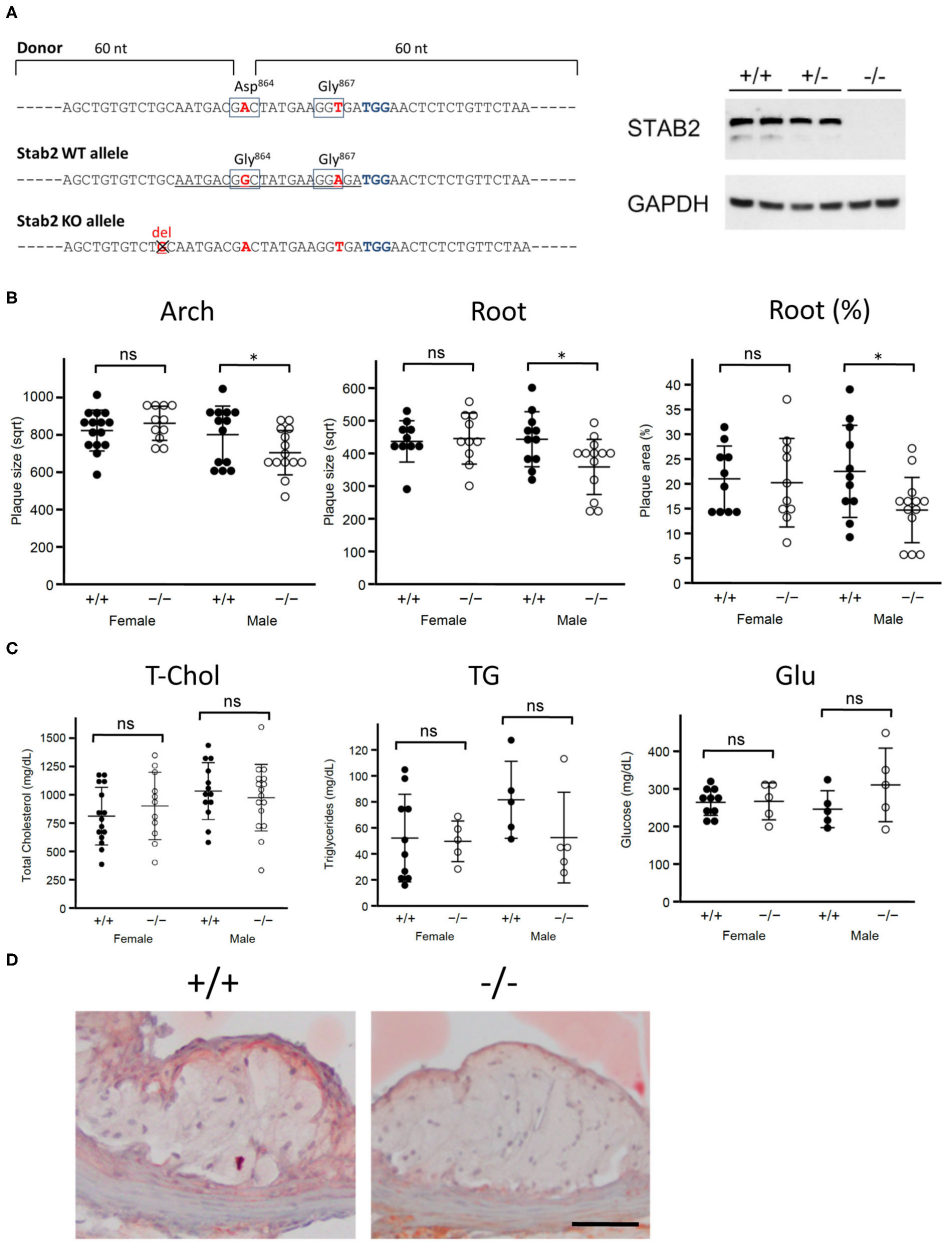
*Stab2*^−/−^*Apoe*^−/−^ mice develop smaller atherosclerotic plaques. **(A)** Generation of *Stab2*^−/−^*Apoe*^−/−^ mice on a C57BL/6 background. Donor sequences carrying two mutations are shown (Top). The guide sequence used in the CRISPR/Cas9 genome editing was underlined (middle). Two mutations were introduced compared to WT allele. Extra one base pair deletion in *Stab2* gene resulted in a knockout allele of *Stab2* (bottom). Complete deficiency of STAB2 protein was confirmed by Western blot using the whole liver lysates from *Stab2*^+/+^, *Stab2*^+/−^ and *Stab2*^−/−^ mice. GAPDH was used as an internal control. **(B)** Comparison of plaque size at the aortic arch (left) and root (middle), and percentage of plaque area vs. vessel area at the root (right) between the control *Stab2*^+/+^*Apoe*^−/−^ and *Stab2*^−/−^*Apoe*^−/−^ mice at 12 weeks old. Mice were fed with a Western-type diet for 8 weeks. Plaque size (μm^2^) was square root transformed (sqrt) for statistical analysis. *n* = 10–16, mean ± s.d., *p < 0.05 vs. *Stab2*^+/+^*Apoe*^−/−^. ns, not significant. **(C)** Plasma lipid and glucose levels in *Stab2*^+/+^*Apoe*^−/−^ and *Stab2*^−/−^*Apoe*^−/−^ mice. *n* = 10–16, mean ± s.d. **(D)** Representative histological sections of the aortic root area from the control *Stab2*^+/+^*Apoe*^−/−^ and *Stab2*^−/−^*Apoe*^−/−^ males at 12 weeks old after fed with a Western-type diet for 2 months. Sections were stained with Sudan IV. Bar = 50 μm.

*Stab2*^−/−^ mice were generated by CRISPR-Cas9 mediated gene editing on the C57BL/6J background in the UNC Animal Models Core. A guide RNA was designed in exon 24 of *Stab2* gene (Figure 1A) originally aiming to generate a mouse line which carries a single G864D mutation. When a plasmid containing the guide RNA and Cas9 sequences was co-injected together with a donor template oligo for homology-directed repair (HDR) into the pronuclei of the fertilized eggs derived from C57BL/6J, eight mice were obtained. Sequencing of the target site identified that seven mice had variable insertion/deletion mutations. A mouse carrying a mutant allele with one base-pair deletion (Figure 1A) was chosen as a knockout line and further mated with B6-*Apoe*^−/−^ mice to generate *Stab2*^+/−^*Apoe*^−/−^ mice on the C57BL/6J background. The lack of STAB2 protein was confirmed by western blotting using a goat polyclonal antibody against STAB2 (M-20, Santa Cruz). *Stab2*^−/−^*Apoe*^−/−^ mice were obtained by mating *Stab2*^+/−^*Apoe*^−/−^ parents, and the littermate *Stab2*^+/+^*Apoe*^−/−^ mice were used as controls. Genotyping of *Stab2* was performed by PCR using the following primers: forward primer to detect wild-type allele; 5'-AGCTGTGTCTGCAATGACGG-3', forward primer to detect the mutant allele; 5'-AGCTGTGTCTGCAATGATGA-3' and the common reverse primer; 5'-GAAGCAGCATGGCAGGTTAT-3'. Genotyping of *Apoe* was performed by quantitative PCR using the following probe and primer sets: wild-type probe: 5'-TGGGAGCAGGCCCTGAACCG-3'; wild-type forward primer: 5'-GAGTGGCAAAGCAACCAACC-3'; wild-type reverse primer 5'-CAGCGCAGGTAATCCCAGAA-3'; knockout probe: 5'-ACCCATGGCGATGCCTGCTTGCCG-3'; knockout forward primer: 5'-GACGGCGAGGATCTCGTCG-3' and knockout reverse primer: 5'-TATGTCCTGATAGCGGTCCG-3'. Because C57BL/6 are relatively resistant to plaque development, *Stab2*^−/−^*Apoe*^−/−^ mice and *Stab2*^+/+^*Apoe*^−/−^ were fed a Western-type diet (42% calories from fat, TD88137, Harlan Teklad) to accelerate atherosclerosis.

### Phenotyping of Mutant Mice

Body weight and plasma levels of lipids and glucose were determined at the indicated age after fasting for 2 to 4 h. Mice were anesthetized with an overdose of avertin (2,2,2-tribromophenol) and perfused with PBS *via* the left ventricle of the heart, followed by perfusion with 4% paraformaldehyde. Atherosclerotic plaque size at the aortic arch and at the aortic root was measured as previously described (10). Briefly, aorta was dissected from the fixed mouse and cleared of fat tissues. Arch lesions were evaluated on the captured images using Image J software. We previously demonstrated a strong correlation between the lesion size measured by this method and the plaque size measured in the cross-sectional aortas (10). The heart containing the aortic root area was sectioned and stained with Sudan IV. Root lesions were also measured on the captured images using Image J software.

### Tail-Vein Injections of ^125^I-HA

Mice were injected *via* the tail vein with 1 mg/kg of ^125^I-HA. After circulation for the indicated times, the mice were euthanized and tissues were measured for radioactivity and mass. The data is presented as CPM/mg tissue.

### Phagocytosis Assay

Phagocytosis assays using isolated peritoneal exudate cells were performed as previously described (9). Briefly, three-month-old male mice were intraperitoneally injected with 1 ml of 4% thioglycollate in 1x PBS. The elicited peritoneal cells were isolated at 3 days after the injection and incubated in 6-well-plates in DMEM supplemented with 10% fetal bovine serum at 37°C for an overnight. Apoptosis was induced in human Jurkat T cells (ATCC) by incubating with 1 μM staurosporine (Sigma) for 3 h. After staining with 100 ng/ml pHrodo™ Red SE (Thermo Fisher Scientific) for 30 min at room temperature, the apoptotic cells were added to the peritoneal cells at 1 × 10^6^ cells per well. After incubating for 60 min at 37°C, cells were detached from the plates by Accutase (EMD Millipore), stained with anti-CD11b-FITC (1:200, Clone M1/70, BD Pharmingen) and 0.5 μM DAPI. Phagocytosis was measured on LSRFortessa (BD Biosciences) and assessed by FlowJo software.

### Plasma Levels of STAB2 Ligands

Plasma concentrations of HA, heparin and vWF were measured with Hyaluronan DuoSet ELISA kit (R&D systems), Unfractionated Heparin ELISA for Rodent Plasma Samples (Lifespan Technologies) and Mouse vWF ELISA Kit (LifeSpan BioSciences), respectively, according to the manufactures' instructions.

### Size of HA

Serum samples of DBA/2J mice were mixed with an equal volume of 100 mM sodium acetate (pH 6.0) and incubated with or without 20 U/ml of Hyaluronidase from *Streptomyces hyalurolyticus* (EMD Millipore) for 2 h at 60°C. Samples were then incubated with 0.5 μg/μl of protease K for 3 h at 60°C, followed by 10 min at 95°C for inactivation of protease K. Nucleic acids were digested with 10 U/μl Benzonase nuclease (Sigma) overnight at 37°C. The samples were precipitated with ethanol and separated by electrophoresis on a 2% agarose gel in TAE buffer. The gel was incubated with 0.005% Stains-All (Sigma) in 30% ethanol overnight and de-stained with 10% ethanol. Select-HA HiLadder and LoLadder (Echelon Biosciences) were used as molecular markers.

### Endocytosis of ^125^I-HA by LSECs

Mouse liver perfusions and LSEC purification was performed as previously described in Cabral et al. with the additional step of depleting Kupffer cells with the use of CD11 magnetic beads (26). Cells were plated on fibronectin-coated 24-well-plates in Roswell Park Memorial Institute (RPMI) medium with 5% serum and incubated for 1.5 h to recover and attach to the plate. The cells were washed and incubated with 1 mg/mL ^125^I-HA for 2 h, washed three times with HBSS and radioactivity was determined with a gamma counter and total cell lysate protein was determined by Bradford Assay.

### Mouse *Stab2* cDNA Isolation

Total RNA was isolated from the liver of 129S6 and DBA/2J mice using RNeasy mini kit (Qiagen) and cDNA was synthesized by SuperScript III (Life Technologies) using random hexamers. The 5' half (3.8 kb) and 3' half (4.2 kb) of the *Stab2* were amplified separately by PCR and cloned into pCMV6-AC-IRES-GFP-Puro vector (OriGene) by the overlap *in vivo* cloning (27, 28). Briefly, the amplified fragments have sequences overlapping with the vector end for 60 bp, and with each other for 300 bp. The mixture of the fragments and vector was directly transformed into Stellar Competent Cells (Takara). The isolated plasmids were sequenced and confirmed to have the full-length cDNAs of *Stab2*^129^ and *Stab2*^*DBA*^. *Stab2* cDNAs carrying each amino acid mutation were made using GeneArt Site-Directed Mutagenesis System (Thermo Fisher Scientific) according to the manufacturer's protocol.

### Stable Transformants of *Stab2* cDNA

Both *Stab2*^129^ and *Stab2*^*DBA*^ cDNAs were transfected into HEK293T cells, and cells stably expressing *Stab2* cDNA were cloned by puromycin selection. To establish cell lines which express similar amounts of the transgene, both 129 and DBA variants were cloned into a pcDNA5/FRT/V5/6xHis-TOPO plasmid (Thermo Fisher Scientific) and stable clones were generated in Flp-In 293 cells (Thermo Fisher Scientific) as previously described (29).

### Direct HA Binding Assay

Flp-In 293 cells stably expressing STAB2 were lysed in 1x PBS containing 0.5% NP-40 and protease inhibitor cocktail. The clarified lysate was combined with 20 μL of a 1:1 slurry of anti-V5 and ^125^I-HA, and incubated for 2 h under slow rotation. The resin was washed 4 times with 1x PBS containing 0.1% NP-40 and evaluated for radioactivity. STAB2 protein was eluted from the resin with Laemmli buffer, separated by 5% SDS-PAGE and evaluated by Western analysis to equilibrate counts per μg of protein.

### Cell Surface and Total Cellular HA Binding Assay

The amount of HA binding by the cell surface and the total cell STAB2 was determined as described previously (30). Briefly, at least three stable clones expressing STAB2 were incubated on ice with 1 μg/mL ^125^I-HA for 1 h at 4°C, with or without 0.055% (w/v) digitonin which permeabilizes the plasma membrane. Cells were washed three times with ice-cold Hanks' balanced salt solution (HBSS), lysed in 0.3 mol/L NaOH, and radioactivity and protein content were determined. The values are expressed as counts per μg of protein.

### Endocytosis of ^125^I-HA by Stab2 Expressing Cells

Endocytosis of ^125^I-HA was performed as described previously (29). Briefly, at least three clones stably expressing STAB2 were plated in 24-well-dishes and allowed to grow for 2 days prior to the experiment. Immediately prior to the experiment, cells were washed and incubated in endocytosis medium (Dulbecco's Modified Eagle's Medium (DMEM) + 0.05% BSA) at 37°C for 1 hour followed by endocytosis medium containing 1 μg/mL ^125^I-HA. At each time point, cells were washed 3 times with ice-cold HBSS, lysed in 0.3 mol/L NaOH, and assessed for radioactivity with a gamma counter. CPM were normalized to the receptor expression levels as assessed by Western blot analysis and expressed as pmol HA per fmol receptor.

### Endocytosis of acLDL

HEK293T cells were grown in DMEM supplemented with 10% fetal bovine serum. *Stab2* cDNA plasmid was transfected into HEK293T cells using FuGENE HD (Promega) following the instructions. Forty-eight h after the transfection, medium was changed to serum-free phenolphthalein-free DMEM, and DiI-labeled acLDL (Alfa Aesar) was added to the cells at the final concentration of 50 μg/ml. Cells were incubated at 37°C for 30 min, washed with PBS and fixed with 4% PFA at room temperature for 10 min. Fluorescent images of the DiI-positive cells were captured by Olympus IX81 microscope (Olympus). Percentages of the DiI-positive cells were determined by FACSAria II (BD Biosciences) and assessed by FlowJo software.

### *In vivo* Clearance of acLDL

One μg/g body weight of DiI-labeled acLDL (Alfa Aesar) in PBS was injected into the anesthetized mice by retro-orbital injection *via* the right eyes. At each time point, blood was collected from the left eyes, and the plasma was transferred to disposable capillaries with 20 μl capacity (#1-000-0200-32, Drummond Scientific Company). Images of the capillaries were captured with automatic exposure times using IX70 fluorescence microscope (Olympus) and SPOT image capture software (SPOT Imaging). Since each exposure time is in inverse proportion to the fluorescence intensity, logarithm of the reciprocal of the exposure time [log_10_ (1/time)] was plotted as the indicator of the fluorescence intensity.

### Statistics

Comparisons between samples were done by *t*-test or one-way analysis of variance (ANOVA) followed by Tukey-Kramer's HSD test. *P* < 0.05 was considered as statistically significant. Data were analyzed using JMP software version 15.2 (SAS Institute) and SigmaPlot 11.2 software (Systat).

## Results

### *Aath5^*DBA*^* Is Slightly Protective Against Atherosclerotic Plaque Development

*Aath5* locus on Chr10 (30-101 Mb) was previously identified as an atherosclerosis QTL. The DBA/2J allele of *Aath5* (*Aath5*^*DBA*^) was associated with smaller plaques, while 129 allele of *Aath5* (*Aath5*^129^) was associated with larger plaques (7). To confirm the effect of *Aath5* on plaque development, we generated a congenic *Apoe*^−/−^ mouse line with *Aath5*^*DBA*/*DBA*^ by backcrossing the genomic region of DBA containing a distal portion of *Aath5* (84.6-93.9 Mb) to 129-*Apoe*^−/−^ for ten generations (Supplementary Figure 1A). The backcrossed region of *Aath5*^*DBA*^ contains *Stab2*^*DBA*^. As expected, plasma concentration of HA was more than 10 times higher in *Aath5*^*DBA*/*DBA*^*Apoe*^−/−^ mice than in male and female control *Aath5*^129/129^*Apoe*^−/−^ mice (Supplementary Figure 1B). Of note, expression of *Stab2*^*DBA*^ was completely suppressed on this 129S6 background because the IAP element interferes with the normal transcription (16). Plasma cholesterol and triglyceride levels in the *Aath5*^*DBA*/*DBA*^*Apoe*^−/−^ mice were slightly lower compared to the control mice, although it was not statistically significant (Supplementary Figure 1C). Plasma glucose levels were comparable between *Aath5*^*DBA*/*DBA*^*Apoe*^−/−^ and the control mice (Supplementary Figure 1C). Atherosclerotic plaque size at the arch area was slightly lower in *Aath5*^*DBA*/*DBA*^*Apoe*^−/−^ mice than in control mice in both males and females, although it was not statistically significant (Supplementary Figure 1D). At the root area, only female *Aath5*^*DBA*/*DBA*^*Apoe*^−/−^ mice showed significantly smaller plaques compared to the control mice (Supplementary Figure 1D). These results support that the backcrossed region of *Aath5*^*DBA*^ confers protection against atherosclerosis. This approach eliminates the potential effects of genes on chromosomes other than Chr10, although it does not eliminate a possibility that more than one gene within the backcrossed region affect atherosclerosis. The effect of each gene could be either suppressing or enhancing plaque development. The result nonetheless confirms that gene(s) within the backcrossed region collectively confers protection and that there is at least one protective gene within the region. The effects of genes within *Aath5*^*DBA*^ that was not carried into the genome of *Aath5*^*DBA*/*DBA*^ mice were eliminated.

### Lack of *Stab2* Is Protective Against Atherosclerotic Plaque Development

To test whether *Stab2* is a modifier of atherosclerosis, we generated *Stab2*^−/−^*Apoe*^−/−^ mice on C57BL/6 background (Figure 1A). Since the C57BL/6 strain is relatively resistant to plaque development compared to the 129S6 strain at the arch area, mice were fed a Western-type diet starting at 4 weeks of age and until 12 weeks of age to enhance atherosclerosis. In males, plaque sizes were smaller in *Stab2*^−/−^*Apoe*^−/−^ mice than in the control mice at both the arch and root areas, while female *Stab2*^−/−^*Apoe*^−/−^ mice did not show significant differences compared to the control mice at either arch or root area (Figure 1B). Plasma total cholesterol and triglyceride levels in the *Stab2*^−/−^*Apoe*^−/−^ mice were not significantly different from the control mice in both males and females (Figure 1C). Plasma glucose levels were also comparable between the two groups (Figure 1C). The histological sections of the aortic root area showed accumulation of Sudan IV-positive foam cells within the plaques in both *Stab2*^+/+^*Apoe*^−/−^ and *Stab2*^−/−^*Apoe*^−/−^ (Figure 1D). These results indicate that the lack of *Stab2* contributes to a small protection against atherosclerosis in males.

### Reduced Clearance of HA From Circulation in *Stab2^−/−^* Mice

**Figure 2:**
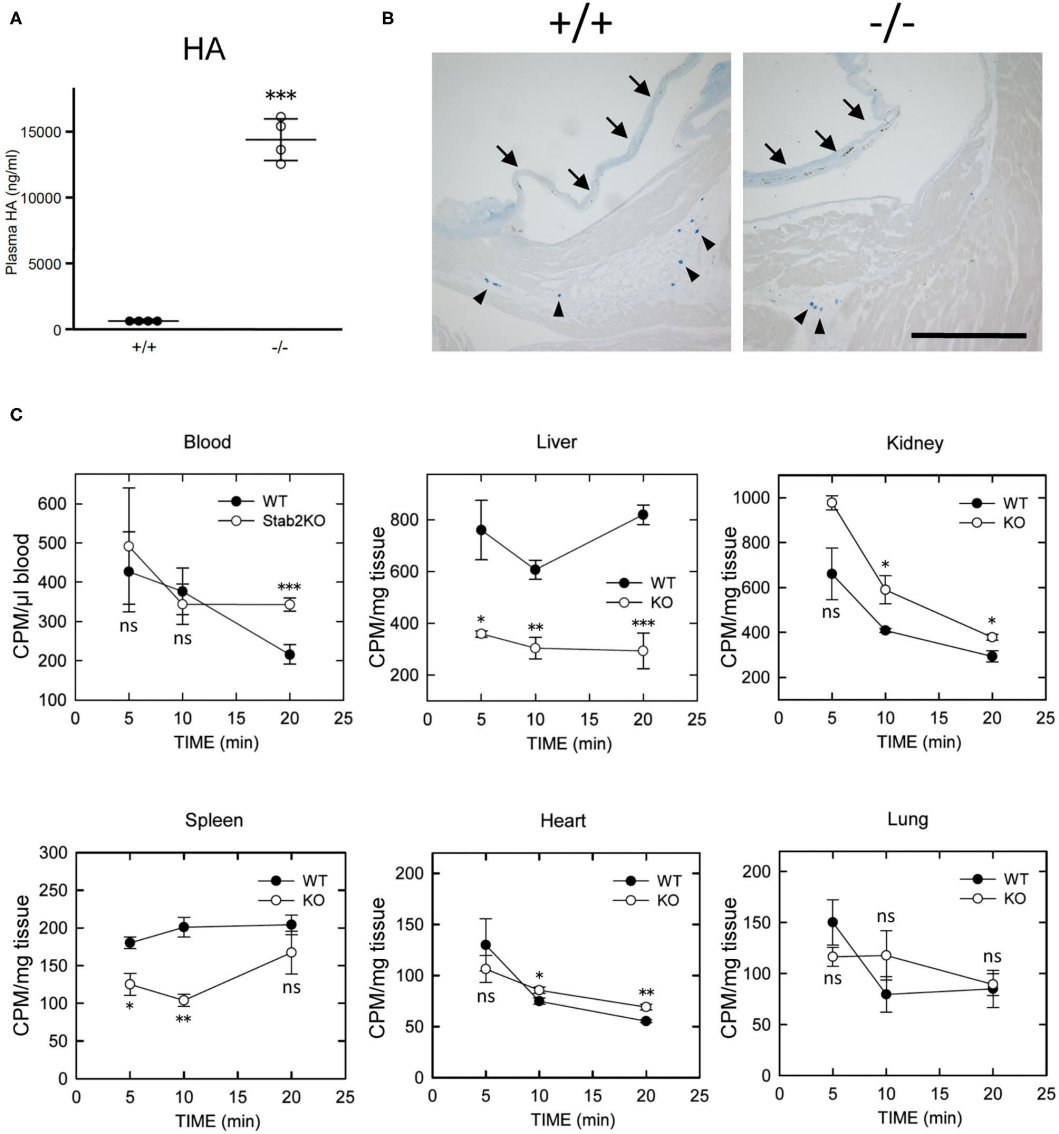
Lack of STAB2 leads to the accumulation of HA in circulation but not in major organs. **(A)** Plasma HA concentrations in *Stab2*^+/+^*Apoe*^−/−^ and *Stab2*^−/−^*Apoe*^−/−^ mice (*n* = 4). Data are mean ± s.d. **(B)** Alcian blue staining of aortic root area of *Stab2*^+/+^*Apoe*^−/−^ and *Stab2*^−/−^*Apoe*^−/−^ mice. Arrows indicate root valve leaflets. Alcian blue-positive mast cells are observed (arrowheads). Bar = 100 μm. **(C)** Distribution of ^125^I-HA in major organs after injection of 1 mg/kg ^125^I-HA *via* tail-vain in *Stab2*^+/+^ (WT) and *Stab2*^−/−^ (KO) mice (*n* = 3; data are mean ± s.d. *p < 0.05, **p < 0.01, and ***p <0.001 vs. *Stab2*^+/+^ mice. ns, not significant).

Consistent with previous reports (18, 20), plasma concentration of HA was elevated by more than 30 times in *Stab2*^−/−^*Apoe*^−/−^ mice compared to the *Stab2*^+/+^*Apoe*^−/−^ mice (Figure 2A). Alcian-blue staining, which detects mucopolysaccharides including HA, showed positive staining in the root valve leaflets and the proximal part of the valves (Figure 2B). Despite the extremely higher plasma HA, the staining pattern in *Stab2*^−/−^*Apoe*^−/−^ mice was comparable to that in the control mice (Figure 2B).

When ^125^I-HA was injected *via* tail vein to wild-type mice, ^125^I-HA in circulation gradually decreased and dropped to less than 50% at 20 min (Figure 2C). In contrast, ^125^I-HA decreased for the first 10 min but became stable in *Stab2*^−/−^ mice. The liver and the spleen of *Stab2*^−/−^ mice showed consistently lower levels of ^125^I-HA than those of wild-type mice. Although more ^125^I-HA was accumulated in the kidney of *Stab2*^−/−^ mice compared to the wild-type, ^125^I-HA decreased to comparable levels as wild-type at 20 min. These results indicate that degradation of HA in the liver and the spleen was inhibited in *Stab2*^−/−^ mice and HA accumulated in circulation. In spite of the high distribution of HA in the liver and the kidney, less ^125^I-HA was detected in the heart and the lung. The distribution patterns of HA in the heart and the lung were similar in wild-type and *Stab2*^−/−^ mice, indicating these are background levels due to residual blood in the organs (Figure 2C).

Collectively, these results suggest that clearance of HA from the liver was reduced in *Stab2*^−/−^ mice, and that HA accumulated in circulation rather than in major tissues/organs including large vessels.

### Other Ligands of STAB2

**Figure 3:**
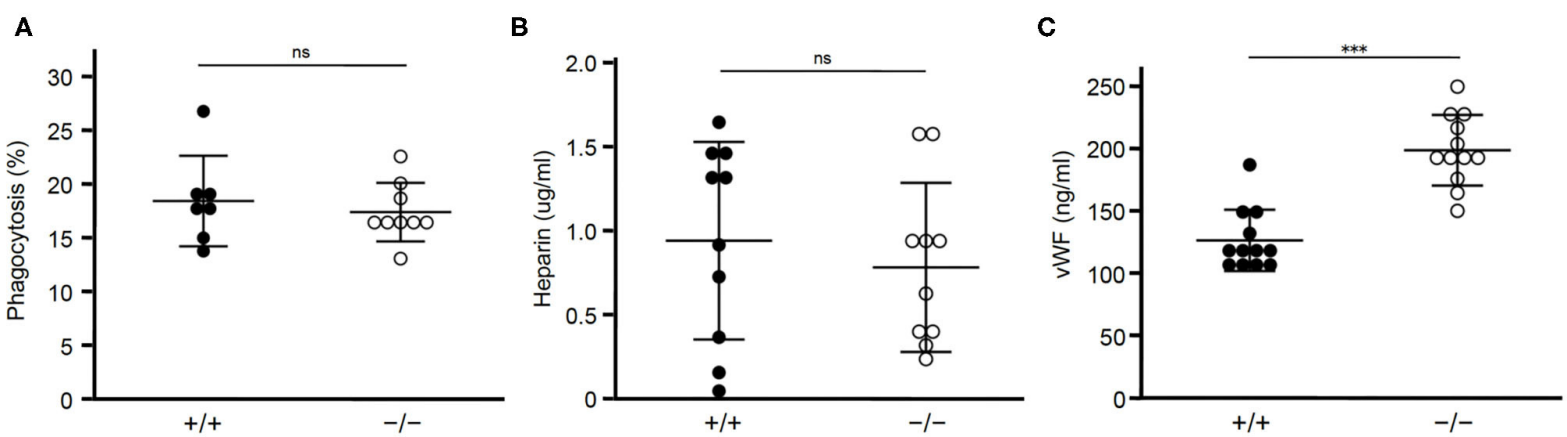
Lack of STAB2 does not have large effects on its ligands other than HA. **(A)** Phagocytosis of apoptotic cells by peritoneal exudate cells isolated from *Stab2*^+/+^*Apoe*^−/−^ (+/+) and *Stab2*^−/−^*Apoe*^−/−^ (–/–) mice. Apoptotic Jurkat T cells labeled with pHrodo Red were added to the peritoneal cells. After the incubation for 1 h at 37°C, cells engulfing the apoptotic cells were detected by flow cytometry (*n* = 7-9). Data are mean ± s.d. **(B,C)** Plasma concentrations of heparin (**B**) (*n* = 10) and vWF **(C**) (*n* = 12) in *Stab2*^+/+^*Apoe*^−/−^ and *Stab2*^−/−^*Apoe*^−/−^ mice. Data are mean ± s.d.

Next we examined whether clearance of STAB2 ligands other than HA were affected by inhibition of *Stab2*. Elimination of apoptotic cells in atherosclerotic plaques plays a critical role in lesion development. STAB2 recognizes phosphatidylserine of apoptotic cells and mediates phagocytosis of those cells (31). We tested the effect of *Stab2* deficiency on phagocytosis using peritoneal exudate cells isolated from *Stab2*^−/−^*Apoe*^−/−^ and the control *Stab2*^+/+^*Apoe*^−/−^ mice. When the isolated peritoneal cells were incubated with apoptotic Jurkat cells, the percentages of macrophages engulfing the apoptotic cells were not significantly different between the two groups. The result indicates that phagocytosis of apoptotic cells is unlikely the primary mechanism involved in the athero-protection in *Stab2*^−/−^*Apoe*^−/−^ mice (Figure 3A).

Heparin, which binds and activates antithrombin, is a ligand of STAB2 (12). Plasma concentrations of heparin were not significantly different between the two groups (Figure 3B). Another ligand of STAB2, von Willebrand Factor (vWF), mediates vascular inflammation and enhances atherosclerosis by stimulating the adhesion of platelets onto the vessels (32). Human GWAS revealed variations of *Stab2* gene are associated with plasma vWF concentration (33). *Stab2*^−/−^*Apoe*^−/−^mice showed approximately 1.6 times higher concentration of plasma vWF compared to *Stab2*^+/+^*Apoe*^−/−^ mice (Figure 3C). This increase was statistically significant but small compared to the elevation of HA, which was 30 times higher in the *Stab2* deficient mice.

Taken together, Stab2 ligands other than HA we tested do not appear to be large effectors of the lack of *Stab2* and do not explain their involvement in the athero-protection in *Stab2*^−/−^*Apoe*^−/−^mice.

### Reduced Clearance of HA in DBA/2J Mice

**Figure 4:**
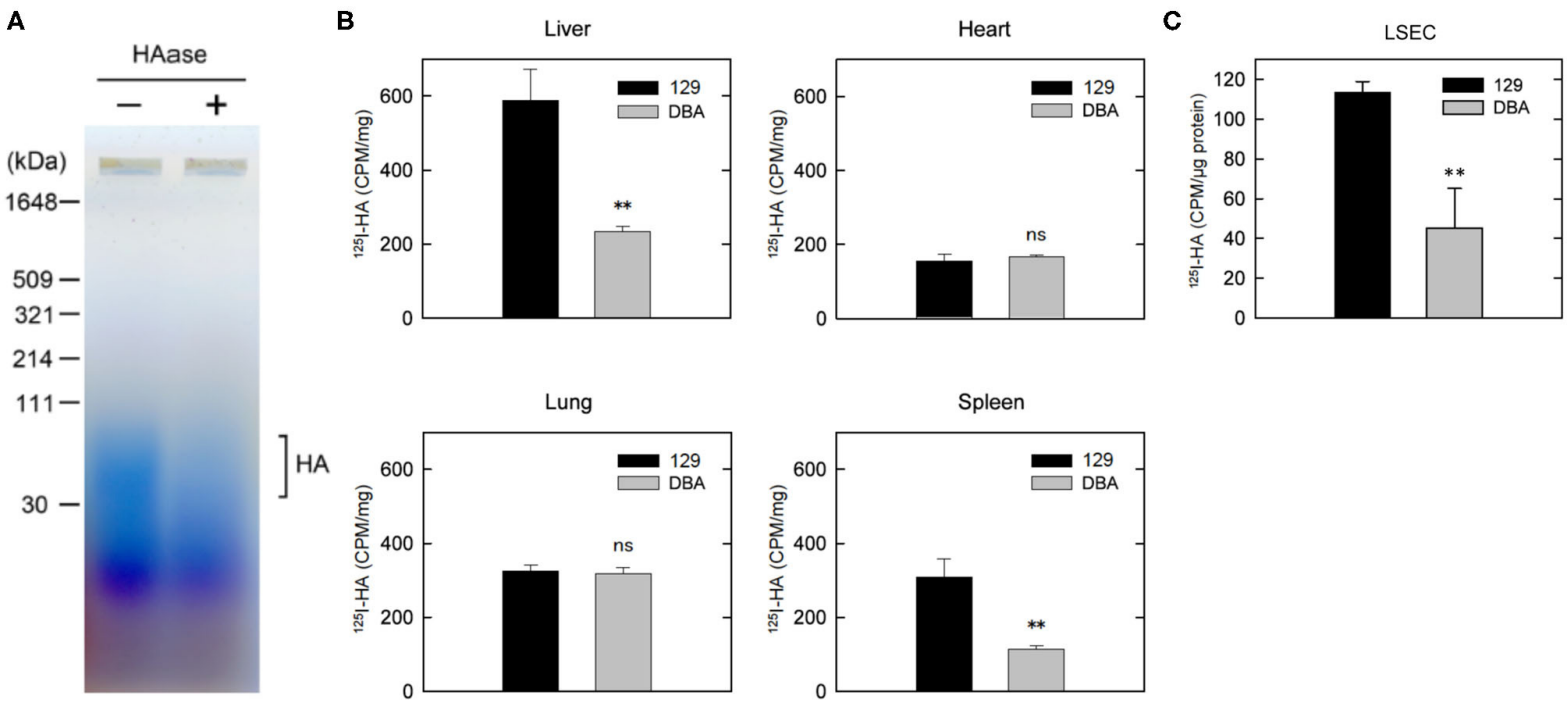
Clearance of HA is disturbed in DBA/2J mice. **(A)** Detection of HA in the plasma of DBA/2J mice. Plasma samples with or without HAase treatment were separated by 2% agarose gel electrophoresis and stained with Stains-All. **(B)** Distribution of ^125^I-HA at 10 min after the tail-vain injection in129S6 and DBA/2J mice (*n* = 3; data are mean ± s.d.). **(C)** Internalization of ^125^I-HA by LSECs isolated from the liver of 129S6 and DBA/2J. LSECs were incubated with medium containing 1 μg/ml ^125^I-HA for 2 h, lysed and assessed for radioactivity. The value (counts per min) was normalized to the amount of protein. The experiment was replicated four times with *n* = 3 for each experiment. Data are mean ± s.d. **p < 0.01 vs. 129. ns, not significant.

We have previously reported that plasma concentration of HA was over 10 times higher in DBA/2J mice than in 129S6 or C57BL/6 mice (7). The size of increased HA in the plasma of DBA/2J mice was approximately 40–100 kDa (Figure 4A). We have also demonstrated that the expression level of STAB2 in LSECs of DBA/2J mice (STAB2^DBA^) was reduced to approximately 35% compared to 129S6 or C57BL/6, and that the promoter region of *Stab2* gene in the DBA allele contains a viral-derived sequence, IAP, which likely interferes with transcription of *Stab2* (16). To confirm that clearance of HA is disturbed in DBA/2J mice, we injected ^125^I-HA (~130 kDa average mass) into 129S6 and DBA/2J mice *via* tail vein, and measured radioactivity in major organs. At 10 min after the injection, the radioactivity of ^125^I-HA was highest in the liver of 129S6, while it was more than 50% lower in that of DBA/2J, suggesting that uptake of ^125^I-HA into the LSECs was reduced in DBA/2J (Figure 4B). The spleen of 129S6 also showed accumulation of ^125^I-HA, although the radioactivity was approximately 50% of the liver. This reflects that STAB2 is also expressed in the SECs in the spleen. Similarly to the liver, the spleen showed significantly lower ^125^I-HA in DBA/2J than in 129S6. The heart and the lung also showed low levels of radioactivity, but no significant differences were observed between 129S6 and DBA/2J. The low levels of radioactivity are considered to be background caused by residual blood in the each organ containing ^125^I-HA. These results support that endocytosis of ^125^I-HA in the liver and spleen was significantly decreased in DBA/2J.

We further isolated LESCs from the livers of 129S6 and DBA/2J and performed endocytosis of ^125^I-HA *ex vivo*. Consistent with the *in vivo* results, endocytosis of ^125^I-HA by the LSECs isolated from DBA/2J mice was more than 50% lower than that of 129 LSECs (Figure 4C). Together, these results confirm that clearance of HA *via* LSECs is reduced in the DBA/2J strain, leading to more than ten times higher concentration of HA in the plasma of DBA/2J mice. However, since the *Stab2* expression in the LSECs of DBA/2J is not completely suppressed, there is a possibility that the STAB2^DBA^ protein contributes to further functional defects due to amino acid alterations specific to DBA/2J.

### Normal Binding and Endocytosis of HA *via* STAB^DBA^ Protein

**Figure 5:**
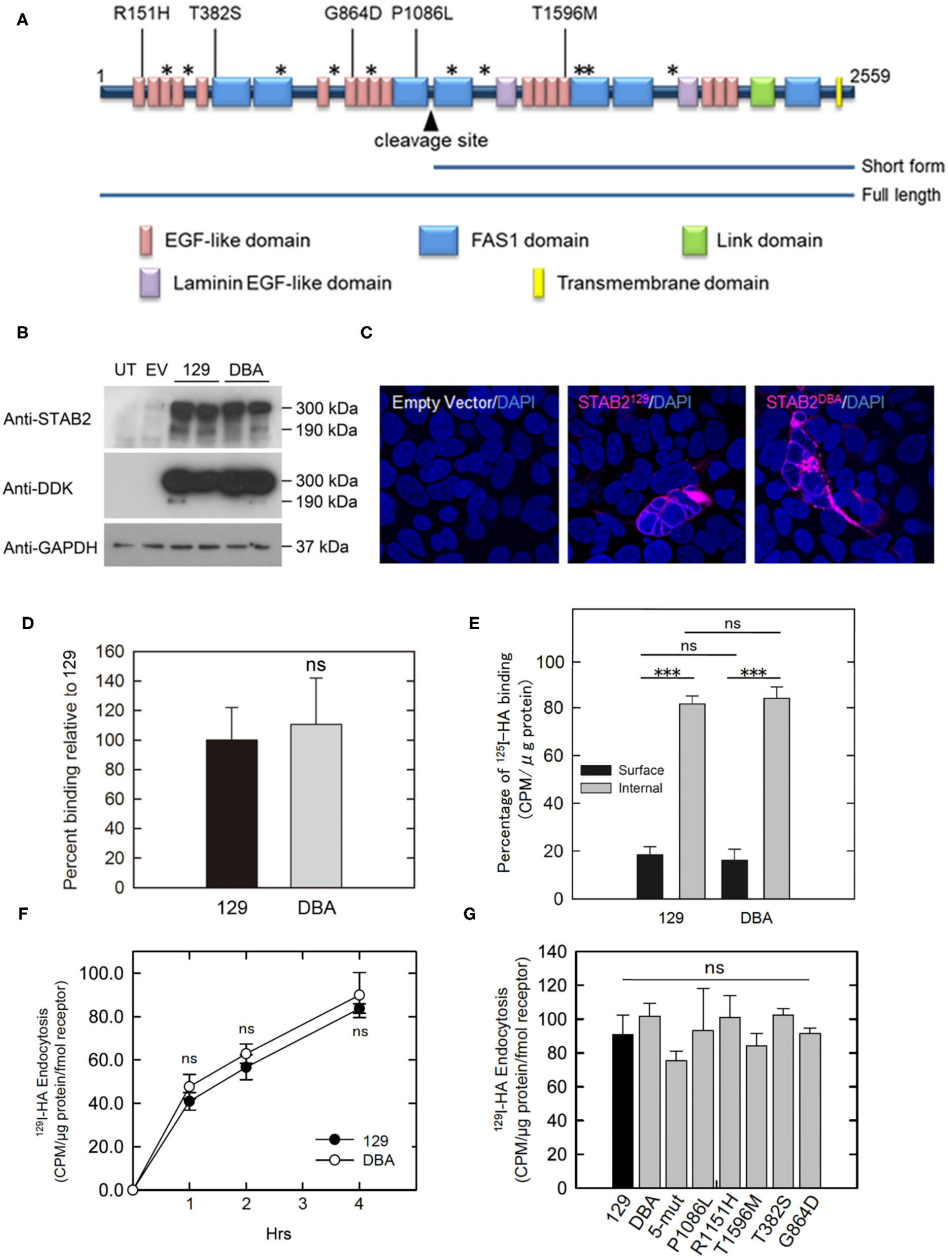
Amino acid changes in STAB2^DBA^ do not affect internalization of HA. **(A)** Domain structure of mouse STAB2. Arrowhead indicates the cleavage site that produces the short form of STAB2. Amino acid (AA) replacements in DBA/2J that were predicted by SIFT program to affect the protein function are shown as [AA in 129]-position-[AA in DBA]. Positions of other AA changes predicted to be benign are indicated as *. **(B)** Expression of STAB2 in the HEK293T cells transfected with the control empty pCMV6-AC-IRES-GFP-Puro vector (EV) or vectors containing *Stab*2^129^ or *Stab*2^DBA^ cDNA. Cells were lysed at 48 h after the transfection and analyzed by western blot using antibodies against STAB2 and the DDK-tag. UT: untransfected HEK293T cells. **(C)** Immunostaining of STAB2 protein (magenta) transiently overexpressed in HEK293T cells detected by anti-DDK antibody. Nuclei were visualized by DAPI staining. **(D)** Binding of ^125^I-HA by STAB2^129^ (129) and STAB2^DBA^ (DBA). Lysates of Flp-In HEK293 cells which express STAB2 tagged with V5 epitope were immunoprecipitated with anti-V5 resin and incubated with 2 μg/ml ^125^I-HA. The radioactivity bound to the anti-V5 resin was measured. The value was normalized to the amount of STAB2 protein. At least three clones were used for each strain. Data are mean ± s.d. **(E)** Distribution of ^125^I-HA bound to STAB2^129^ and STAB2^DBA^ on the cell surface and in the intracellular compartments. STAB2-expressing HEK293T cells were incubated with ^125^I-HA on ice without (Surface) or with (Total) digitonin, which permeabilize the cells and allow ^125^I-HA access to intracellular pools of STAB2. Cells were lysed and the radioactivity was assessed. Internal binding (%) was calculated as [100 - Surface binding (%)]. Four clones of 129 and three clones of DBA were used. At least four experiments were done for each clone and the values were averaged. Data are mean ± s.d. **(F)** Internalization of ^125^I-HA by STAB2^129^ and STAB2^DBA^. STAB2-expressing HEK293T cells were incubated with medium containing 1 μg/ml ^125^I-HA for the indicated times, lysed and assessed for radioactivity. The value was normalized to the amount of STAB2 protein determined by Western blotting. Four clones of 129 and three clones of DBA were used. Experiments were repeated five times and average values are shown. Data are mean ± s.d. **(G)** Internalization of ^125^I-HA by STAB2 proteins carrying each DBA2/J-specific amino acid change (P1086L, R151H, T1596M, T382S and G864D) or a mutant STAB2 protein where N-terminus region (1-927 AA) of STAB2^129^ was changed to that of DBA2/J (5-mut). STAB2-expressing HEK293T cells were incubated with medium containing 1 μg/ml ^125^I-HA for 4 h and endocytosis of ^125^I-HA were assessed as in **(F)**. *n* = 4. Experiments were repeated five times and average values are shown. Data are mean ± s.d. ***p < 0.001 vs. 129. ns, not significant.

Mouse STAB2 protein has a large (2,464 amino acids) extracellular region composed of multiple conserved domains indicative of protein-protein interaction. Although no alteration was found in the HA-binding link domain of STAB2^DBA^, STAB2^DBA^ differs from STAB2^129^ by 15 amino acid residues, five of which: R151H, T382S, G864D, P1086L and T1596M, are potentially deleterious substitutions according to the SIFT prediction program (7). These amino acids are located in the EGF-like domains or in FAS domains within the extracellular region, and could affect polymerization or interaction with other receptors (Figure 5A). To test the effects of these amino acid changes on the functions of STAB2, we constructed expression vectors containing individual *Stab2* cDNAs isolated from the livers of 129S6 and DBA/2J. The vector provides a DDK epitope tag at the C terminus of the STAB2 protein. A previous report by Harris *et al*. had shown that the C-terminal tag does not affect the expression and function of STAB2 (34). When the same amount of cDNA was overexpressed in human embryonic kidney 293T (HEK293T) cells, comparable amounts of STAB2^129^ and STAB2^DBA^ proteins were detected by Western blot at approximately 300 kDa and 175 kDa as expected (Figure 5B). The protein is localized on the cell surface, indicating that membrane trafficking of STAB2^DBA^ is not affected by the amino acid alterations (Figure 5C).

To compare the binding capacity of STAB2^129^ and STAB2^DBA^ to HA, Flp-In 293 cells stably expressing each STAB2^129^ or STAB2^DBA^ tagged with C-terminal V5 were lysed, immunoprecipitated with anti-V5 resin, and incubated with ^125^I-HA. When normalized to the amount of protein levels, binding of HA to the immunoprecipitated STAB2^DBA^ was not significantly different from STAB2^129^ (Figure 5D). To further examine HA binding to STAB2^129^ and STAB2^DBA^ in live cells, STAB2-expressing HEK293T cells were incubated with ^125^I-HA on ice with or without 0.055% digitonin. Since digitonin permeabilizes the cells and allows ^125^I-HA access to intracellular pools of STAB2, HA binding in the intracellular compartments as well as on the cell surface can be detected, which is defined as total ^125^I-HA binding. Despite that each cell line expresses varied amounts of STAB2, the percentage of ^125^I-HA bound to the cell surface was 18.4 ± 1.4% compared to internal binding of 81.6 ± 1.4% in STAB2^129^ cells. In STAB2^DBA^ cells, the cell surface binding was 16.1 ± 2.2% while internal binding was 83.9 ± 2.2%. Thus, there is no significant difference in the trafficking of the receptor and HA-binding activity in STAB2^129^ and STAB2^DBA^ expressing cells (Figure 5E).

The endocytosis of ^125^I-HA by cells overexpressing STAB2^129^ and STAB2^DBA^ were also comparable (Figure 5F). Moreover, cells overexpressing STAB2 which carry each of the five amino-acid mutations did not show significant difference in endocytosis of ^125^I-HA (Figure 5G). Therefore, these amino acid changes in STAB2^DBA^ are unlikely to contribute to the high plasma concentration of HA in DBA/2J mice. These data support our previous finding that the reduced expression at the transcriptional level of *Stab2* in LSECs is the primary contributor to the elevation of plasma HA.

### Clearance of acLDL Is Not Affected in DBA/2J

**Figure 6:**
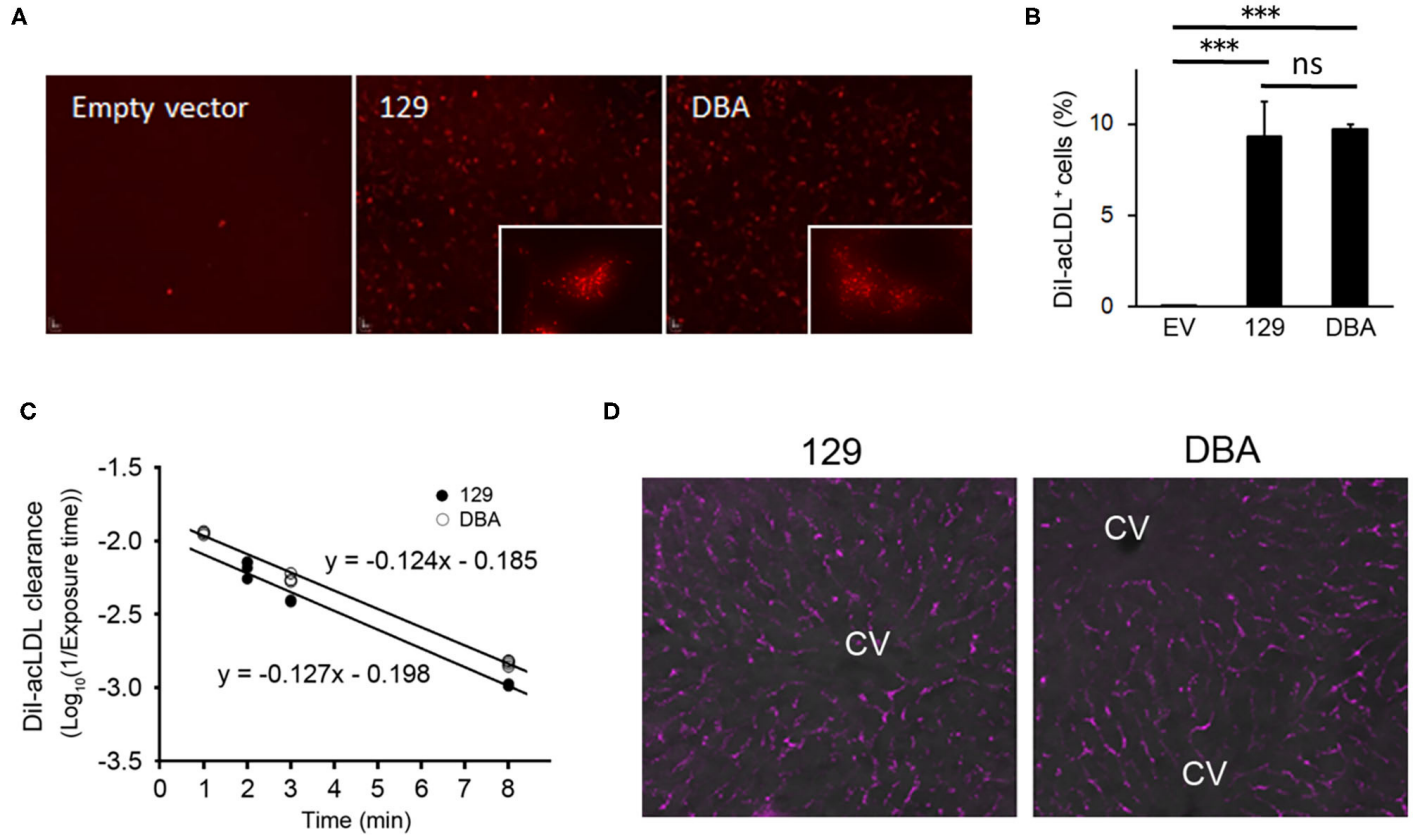
Amino acid changes in STAB2^DBA^ do not affect internalization of acLDL. **(A)** Endocytosis of DiI-acLDL by the STAB2-expressing HEK293T cells. Cells were incubated with 50 μg/ml DiI-acLDL at 37°C for 30 min. Images were captured after washing with PBS and fixation with 4% PFA. The inserts show the punctate pattern of intracellular distribution. **(B)** Percentage of DiI-acLDL positive cells. STAB2-expressing HEK293T cells were incubated with 50 μg/ml DiI-acLDL at 37°C for 15 min and analyzed by flow cytometry. Data are mean ± s.d. The *** symbol indicates the value *p* < 0.001. ns, not significant. **(C)** Clearance of DiI-acLDL *in vivo*. 129S6 and DBA/2J mice were intravenously injected with 1 μg/g body weight of DiI-acLDL. Blood was collected at the indicated time points and fluorescence intensity in the plasma was determined (*n* = 4 for each strain). **(D)** Fluorescent images of the liver from the 129S6 and DBA/2J mice 9 min after the injection of DiI-acLDL. DiI-acLDL particles were distributed along the liver sinusoids similarly in both strains. CV; central vein.

Since modified LDL, another ligand of STAB2 (35), is known to enhance atherosclerotic plaque development, we examined whether the allelic differences of *Stab2* influence clearance of acetylated low-density lipoprotein (acLDL). When incubated with culture medium containing DiI-labeled acLDL, cells expressing STAB2^129^ showed increased uptake of DiI-acLDL compared to the control cells transfected with the empty vector, indicating that the cDNA produces functional STAB2 proteins (Figure 6A). Uptake of acLDL by STAB2^DBA^ was not significantly different from that by STAB2^129^ (Figures 6A,B).

To examine the effects of the allelic differences of *Stab2* on the clearance of acLDL *in vivo*, DiI-labeled acLDL was intravenously injected to 129S6 and DBA/2J mice and its clearance from the circulation was detected by measuring fluorescence intensity of the plasma. The injected acLDL was rapidly cleared in 129S6 mice (Figure 6C). Despite the reduced amount of STAB2 in the LSECs, DBA mice did not show any noticeable delay in the clearance of acLDL (Figure 6C). At 9 min post-injection, the DiI-acLDL was distributed along the sinusoids in the liver, and the distribution patterns and intensities of the DiI-acLDL were not significantly different between 129S6 and DBA/2J (Figure 6D). The reduced STAB2 expression in DBA/2J does not appear to affect the clearance of acLDL *in vivo*, probably because the uptake of acLDL is also mediated by other scavenger receptors, including SR-A, SR-B and STAB1.

Taken together, our data suggest that the increased plasma of mid-sized HA is the major reason that *Stab2* deficiency leads to small protection from atherosclerotic plaque development.

## Discussion

Our previous QTL analyses revealed two major QTLs, *Aath4* on Chr 2 and *Aath5* on Chr 10, where the DBA allele of *Aath4* confers susceptibility to atherosclerosis, whereas, DBA of *Aath5* is protective (7, 8). DBA/2J strain is more susceptible to atherosclerosis compared to C57BL/6 and 129S6, since the protective effect of *Aath5* is not large enough to overcome the effect of *Aath4*. Still, dissecting those factors is a necessary step to better understand the whole genetic structure of a common disease like atherosclerosis that consists of many little factors. Our study using the backcrossed mice and the gene-edited mice demonstrated that *Stab2*, a candidate of *Aath5*, is likely a modifier of atherosclerosis, but the effect can be easily flipped depending on the genotypes of other loci with greater influential effects.

While both male and female *Aath5*^*DBA*/*DBA*^*Apoe*^−/−^ mice tend to show smaller plaques, only male *Stab2*^−/−^*Apoe*^−/−^ mice showed statistically significant protection. The male-specific phenotype of *Stab2*^−/−^*Apoe*^−/−^ mice is consistent with our previous observation that *Aath5* was detected in F2 males of the DBA/2J x 129S6 cross (7). Although there is no direct evidence that explains the male-only phenotype in the *Stab2*^−/−^*Apoe*^−/−^, many sex-dependent factors, such as sex hormones, are known to influence atherosclerosis, vessel diameter and blood pressure (36). Female *Stab2*^−/−^*Apoe*^−/−^ could be protected by the combination of these factors. The phenotype in *Aath5*^*DBA*/*DBA*^*Apoe*^−/−^ mice could be affected by other gene(s) which also underlie *Aath5*. The background of *Aath5*^*DBA*/*DBA*^*Apoe*^−/−^ mice is 129S6, while *Stab2*^−/−^*Apoe*^−/−^ mice are on a C57BL/6 background, which might have introduced the difference as well. Furthermore, although our original QTL analyses found the athero-protection was restricted at the aortic arch area and not at the aortic root (7, 8), the protection was not strictly limited to the aortic arch in the congenic *Aath5*^*DBA*/*DBA*^*Apoe*^−/−^ (129S6) and in *Stab2*^−/−^*Apoe*^−/−^ (C57BL/6) mice. This suggests a possibility that the many other loci are influencing to the atherogenesis at the aortic root area more than in aortic arch area, limiting the ability to detect small effects by the QTL analyses.

In the current work we explored several possible mechanisms by which the lack of *Stab2* could lead to the protection against atherosclerosis. In humans, there is a report that *Stab2* is expressed in atherosclerotic plaques, although the function of *Stab2* is unclear (17). Our previous microarray experiments showed that the expression of *Stab2* in macrophages was almost undetectable in mice (7). Furthermore, our data presented here show that phagocytosis of apoptotic cells was comparable between macrophages isolated from the wild-type and *Stab2*^−/−^*Apoe*^−/−^mice (Figure 3A). Our data, thus suggest that the influence of *Stab2* on the phagocytosis of apoptotic cells by macrophages in atherosclerotic plaques is negligible.

The major ligand of STAB2 is HA, and consistent with previous reports (18, 20), plasma HA concentration was more than 30 times higher in *Stab2*^−/−^*Apoe*^−/−^ mice than in the control *Stab2*^+/+^*Apoe*^−/−^mice (Figure 2A). HA is an unbranched polysaccharide consisting of alternating D-glucuronic acid and N-acetyl-D-glucosamine residues. According to the molecular weight, HA is classified into oligo HA (o-HA, <10 kDa), low to medium molecular weight HA (LMW- to MMW-HA, 10–1,000 kDa) and high molecular weight HA (HMW-HA, >1,000 kDa) (37, 38). HMW-HA can provide increased viscoelasticity to tissue fluids such as vitreous humor and synovial fluid (39). HMW-HA is also important in the cellular microenvironments, modulating cell mobility, immune cell differentiation, adhesion and activation (40). The breakdown of HMW-HA, catalyzed by hyaluronidase activity in tissues, initiates turnover of HA, which enters the lymphatic system and is further degraded in lymph nodes (41). The degraded HA in circulation is endocytosed by LSECs, and is degraded to very short oligos and monosaccharides (42).

Several studies indicate that HA is anti-atherogenic. In a cell culture system, HMW-HA (average ~4,000 kDa) significantly inhibited smooth muscle cell (SMC) migration induced by platelet-derived growth factor-BB (PDGF-BB) (43). *In vivo* experiments also showed that pharmacological inhibition of HA synthesis by 4-methylumbelliferone (4-MU) accelerated plaque development in *Apoe*^−/−^ mice and increased macrophage recruitment to the plaque (21). Furthermore, daily subcutaneous administration of HMW-HA significantly reduced the intima-media ratio and intimal macrophage content after balloon injury of the common carotid artery in cholesterol-fed rabbits (22). A recent study also demonstrated that injection of nanoparticles that are aggregates of HA stabilized plaques in *Apoe*^−/−^ mice (44). These results collectively suggest that HA in circulation is anti-atherogenic.

On the other hand, in atherosclerosis, HA is an abundant component of plaque matrix (45). Expression of HA synthases as well as hyaluronidases increase in plaque during inflammation, and accumulation of fragmented HA in the plaque matrix stimulates inflammation (46, 47). Inhibition of HA synthesis by 4-MU reduced neointimal hyperplasia in femoral arteries of mice after injury (23). The authors reported that, through binding to the active form of CD44 or other receptors, LMW-HA mediates leukocyte adhesion and extravasation, as well as SMC migration and proliferation following vascular injury (23). In addition, SMC-specific overproduction of HA promoted the development of aortic atherosclerosis in *Apoe*^−/−^ mice (24). Expression of HA synthase 2 (HAS2) in medial SMC-promoted SMC proliferation and migration (23). Moreover, HA synthase 3 (HAS3) knockout mice developed smaller atherosclerotic plaques (47). These results suggest that HA in the plaque ECM could be pro-atherogenic.

The contradictory results could be due to the size of HA, location of HA accumulation and the stages of atherosclerosis. There is also a potential contamination of endotoxin or other bioactive molecules in animal-derived HA for *in vitro* work, which could affect proper evaluation of the HA-intrinsic effects (48). In our analysis, the size of accumulated HA in the plasma of DBA/2J was estimated at approximately 40–100 kDa, which is comparable to that of 129S6 mice. Despite the extremely high concentration of HA in plasma, accumulation of HA in tissues including the aortic wall was not apparent in DBA/2J and *Stab2*^−/−^ mice, suggesting that the effects of STAB2 inhibition on the SMC-derived HA within the plaque are considered to be small.

HA is not the only ligand for STAB2-mediated clearance. Over twenty ligands including HA have been reported, although unlike HA, many of them are also cleared by other receptors (49). For example, modified LDLs (oxLDL, acLDL) are cleared by macrophages *via* several receptors such as CD36, SR-A and SR-B (50). Plasma lipid levels of *Stab2*^−/−^*Apoe*^−/−^ mice were comparable to the control mice, indicating that clearance of modified LDL is unlikely the underlying mechanism. In addition to HA, chondroitin sulfate also constitutes endothelial glycocalyx and could be anti-atherogenic (51). Chondroitin sulfate competes with HA binding on STAB2, although it could be cleared *via* other scavenger receptors including STAB1 (13).

Von Willebrand factor (vWF) and Factor VIII (FVIII) also bind to STAB2. Genetic associations of *Stab2* and vWF and FVIII were suggested by several genome wide association studies (GWAS). A recent report has demonstrated that vWF binds to STAB2 protein and is cleared by LSECs in humans (15). The GWAS Catalog lists significant association of SNPs in or near the STAB2 gene with circulating vWF and FVIII levels, and recent human and mouse studies indicate Stab2 is involved in venous thromboembolism (33, 52, 53). Increased plasma levels of vWF and FVIII have been shown to enhance platelet recruitment to endothelial cells (32). Since platelet-endothelial interaction is a contributing factor in atherosclerosis, STAB2 could affect lesion development *via* vWF/FVIII. Although direct binding of mouse vWF and mouse STAB2 was not observed, STAB2 might affect stability of vWF/FVIII indirectly *via* the accumulation of heparin, which binds to and inhibits vWF, since heparin also binds STAB2 (12). Our result shows that plasma vWF levels were 1.6 times higher in *Stab2*^−/−^*Apoe*^−/−^ mice than in the control mice. Although the elevation of vWF was not as dramatic as HA, which was more than 30 times higher, the accumulation of vWF could somewhat enhance atherosclerosis reducing the athero-protective effects by other ligands in *Stab2*^−/−^*Apoe*^−/−^ mice. Being a large molecule with multiple domains for protein-protein interactions, STAB2 could interact with other clearance receptors for various glycosylated molecules (15). We also note that CLEC4M and SCARA5 are also clearance receptors for FVIII and their interactions with STAB2 are not known at this time (54, 55). These mechanisms need to be addressed in a future study.

Our data demonstrated that DBA-specific amino acid replacements on STAB2, despite being predicted to be potentially harmful replacements, did not alter the internalization of HA. The result is consistent with the fact that all five non-synonymous mutations are outside of the HA-binding link domain that controls internalization of HA. The result instead strongly supports our previous suggestion that reduced transcription of *Stab2* is the primary cause of dysfunction of STAB2 in DBA (16).

In summary, our results indicate that *Stab2* is likely a modifier gene of atherosclerosis. The protective effects we observed were small, and probably affected by other factors. Additionally, there are technical limitations in detecting minor differences in the phenotypes due to animal-to-animal variations. Nevertheless, we point out that all four independent sets of crosses (two previous QTL analyses and current congenic as well as *Stab2*^−/−^*Apoe*^−/−^ mice) showed small but significant protective effects of the reduced STAB2 protein. In no case did we observe significant effects in an opposite direction. Further studies are necessary to determine if the direct effector(s) among the many ligands of STAB2, other than HA, affect plaque formation.

## Data Availability Statement

The original contributions presented in the study are included in the article/Supplementary Material, further inquiries can be directed to the corresponding authors.

## Ethics Statement

The animal study was reviewed and approved by Institutional Animal Care and Use Committees of the University of North Carolina at Chapel Hill and the University of Nebraska.

## Author Contributions

YK, NM-S, and EH designed the experiments. YK, CC, AL, XS, SH, PH, JW, JH, FL, NM-S, and EH performed the experiments. YK, NM-S, and EH analyzed the data. YK, NM-S, and EH wrote the article. All authors contributed to the article and approved the submitted version.

## Funding

This research was supported by a National Institutes of Health (NIH) grants HL042630 and HL049277 to NM-S and HL130864 to EH. The UNC Histology Core and Flow Cytometry Core Facility are supported in part by P30 CA016086 Cancer Center Core Support Grant to the UNC Lineberger Comprehensive Cancer Center. The research was also in part supported by NIH HL150536 grant to XS.

## Conflict of Interest

The authors declare that the research was conducted in the absence of any commercial or financial relationships that could be construed as a potential conflict of interest.

## Publisher's Note

All claims expressed in this article are solely those of the authors and do not necessarily represent those of their affiliated organizations, or those of the publisher, the editors and the reviewers. Any product that may be evaluated in this article, or claim that may be made by its manufacturer, is not guaranteed or endorsed by the publisher.
