# Supplementary material for: Reduction of Stabilin-2 Contributes to a Protection Against Atherosclerosis
Source: Front Cardiovasc Med. 2022 Mar 11;9:818662. doi: 10.3389/fcvm.2022.818662 (PMC8963368; doi:10.3389/fcvm.2022.818662)
Supplement: Supplementary file 1 [file Data_Sheet_1.docx]

**Figure S1. *Aath5^DBA/DBA^Apoe^−/−^* mice develop smaller atherosclerotic plaques.**

(A) Haplotype maps of Chr 10 taken from the Mouse Phylogeny Viewer (http://msub.csbio.unc.edu/#viewer). 129S6 sequence is colored in green (top); genomic regions where 129S6 share the same sequences as C57BL/6 are shown in green, and C57BL/6-specific sequences are highlighted with purple (middle); genomic regions where all the three strains share the same sequences are shown in green, regions where DBA/2J share the same sequences as 129S6 but differs from C57BL/6 are shown in purple, and DBA/2J-unique regions are colored in peach (bottom). The backcrossed region in the congenic strain Aath5DBA/DBA (red bar) and the location of some of the candidate genes (black arrows) are indicated. (B) Plasma HA concentrations in the control 129-*Apoe^−/−^* and *Aath5^DBA/DBA^Apoe^−/−^* mice (n = 3). Data are mean ± s.d. (C) Plasma levels of total cholesterol, triglyceride and glucose in the control 129-*Apoe^−/−^* and *Aath5^DBA/DBA^Apoe^−/−^* mice. n = 14-20. Data are mean ± s.d. (D) Comparison of plaque size at the aortic arch (left) and root (right) between the control 129-*Apoe^−/−^* and *Aath5^DBA/DBA^Apoe^−/−^* mice at 4 months old. Plaque size (μm^2^) was square root transformed (sqrt) for statistical analysis. n = 14-20. Data are mean ± s.d. *p <0.05 vs. control mice. ns, not significant.
