# Supplementary figures and images for: Reduction of Stabilin-2 Contributes to a Protection Against Atherosclerosis
Source: Front Cardiovasc Med. 2022 Mar 11;9:818662. doi: 10.3389/fcvm.2022.818662 (PMC8963368; doi:10.3389/fcvm.2022.818662)

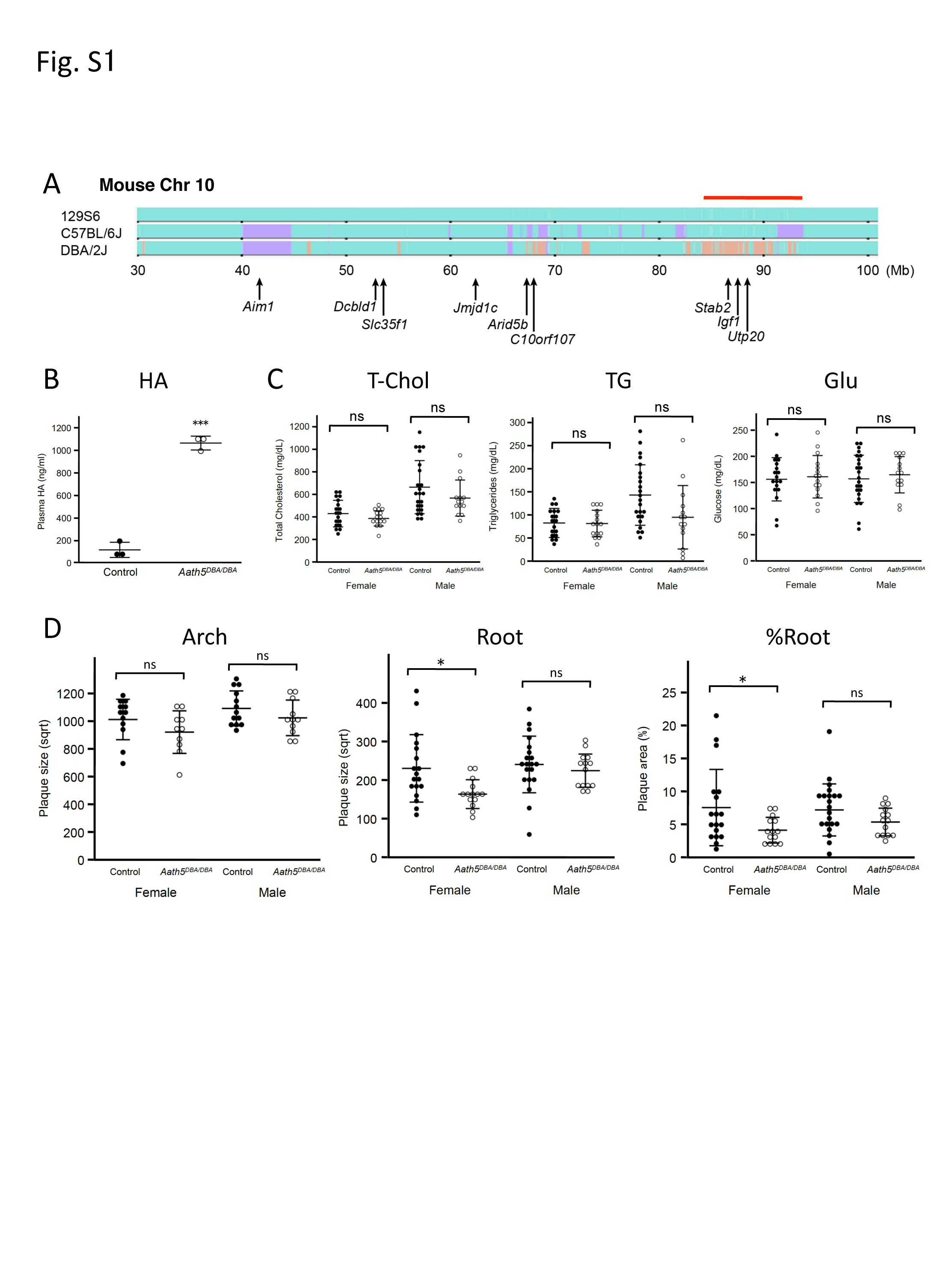

Supplement: Supplementary file 2 [file Image_1.TIF]
